# Complementary effects of coenzyme Q10 and *Lepidium sativum* supplementation on the reproductive function of mice: An experimental study

**DOI:** 10.18502/ijrm.v19i7.9471

**Published:** 2021-08-16

**Authors:** Fatemeh Rahimi Asl, Maryam Khosravi, Ramin Hajikhani, Jalal Solati, Hossein Fahimi

**Affiliations:** ^1^Department of Biology, Islamic Azad University North Tehran Branch, Tehran, Iran.; ^2^Department of Biology, Faculty of Biological Sciences, Islamic Azad University North Tehran Branch, Tehran, Iran.; ^3^Department of Biology, Karaj Branch, Islamic Azad University, Karaj, Iran.; ^4^Department of Genetics, Faculty of Advanced Science and Technology, Tehran Medical Sciences, Islamic Azad University, Tehran, Iran.

**Keywords:** Lepidium sativum, Coenzyme Q10, Infertility, Male reproductive function.

## Abstract

**Background:**

Coenzyme Q10 (CoQ10) and *Lepidium sativum* (LS) have therapeutic effects on infertility.

**Objective:**

To evaluate the combined effects of LS and CoQ10 on reproductive function in adult male NMRI mice.

**Materials and Methods:**

Eighty three-months-old male mice (35–40 gr) were divided into four groups (n = 10/each): control (treated with water), CoQ10-treated (200, 300, and 400 mg/kg/body weight), LS-treated (200, 400, 600 mg/kg/body weight), and co-treated (LS [600 mg/kg/body weight] + CoQ10 [200 mg/kg/body weight]) groups. Serum testosterone, luteinizing hormone, follicle-stimulating hormone, and gonadotropin realizing hormone (GnRH) levels were measured using ELISA method. The sperm quality was assessed using Sperm Class AnalyzerⓇ (SCA) CASA system and GnRH mRNA expression levels were evaluated by real-time polymerase chain reaction.

**Results:**

The number of sniffing and following behavior was significantly higher in LS-treated (400 and 600 mg/ml/body weight) groups than the control group (p = 0.0007 and p = 0.0010, respectively). The number of mounting and coupling behaviors was significantly higher in the CoQ10 (300 and 400 mg/ml/body weight)-treated animals than the control group (p = 0.0170 and p = 0.0006, respectively). Co-treatment of CoQ10 (200 mg/ml/body weight) and LS (600 mg/ml/body weight) significantly increased all aspects of sexual behaviors as well as the levels of serum testosterone (p = 0.0011), luteinizing hormone (p = 0.0062), and follicle-stimulating hormone (p = 0.0001); sperm viability (p = 0.0300) and motility (p = 0.0010); and GnRH mRNA levels (p = 0.0016) compared to the control group.

**Conclusion:**

The coadministration of CoQ10 and LS significantly improves the activity of the hypothalamic-pituitary-gonadal axis and enhances the reproductive parameters in adult male mice.

## 1. Introduction

Chemical medicines used to treat infertility problems are usually followed by side effects, however, herbal medicines have shown to improve sperm quantity and quality as well as testicular function with low side effects (‎1–3). *Lepidium sativum* (LS), sometimes referred to as garden cress is a herbal medicine with anti-infertility activity (‎4). It is a fast-growing annual herb from the *Brassicaceae* family that can reach a height of 50 cm (‎5). LS seeds oil contains a considerable amount of sinapic acid and sinapine, which modulate sex steroids metabolism and act on male reproductive system to improve semen parameters and sperm function (‎6). LS extract are used in many countries for the treatment of various medical conditions including diabetes (‎7), hypertension (‎8), and renal diseases (‎9). Other species of Lepidium such as *Lepidium meyenii* has been reported to have improving effects on sperm count and motility and on sexual behavior in male animals (‎10,‎ 11). It has been shown that plants in the *Brassicaceae *family can elevate sexual desire in healthy menopause women by acting on the hypothalamus-pituitary-gonad axis (‎12). Experimental data have revealed the improving effects of LS on the epididymis morphology in a diabetic rat model (‎‎4) and gonadotropin secretion in rabbits (‎10). Other key factors affecting male fertility are reactive oxygen species (ROS). Under normal physiologic conditions, a slight amount of ROS is produced by sperm cells. However, the excess production of ROS by sperms can cause DNA damage, motility decrease, and sperm membrane dysfunction (‎‎13). The balance between production and destruction of ROS is essential for sperm motility and male fertility (‎14) and hence, the medicines and nutrients that are able to improve this balance can improve male fertility. Antioxidants such as coenzyme Q10 (CoQ10) have been shown to inhibit ROS and prevent oxidative DNA damage (15, ‎‎16).

Although a large body of experimental studies have been carried out to investigate the antioxidant, antimicrobial, and anti-inflammatory characteristics of oil extracted from the seeds of LS, few studies have reported the anti-infertility effects of LS seed oil. There are also very few, if any, reports on the synergistic effects of LS seed extract and CoQ10 on improving the male reproductive system function. Therefore, this study was designed to determine the combined effects of LS and CoQ10 on the reproductive function in adult male NMRI mice and the findings will add new approach in the treatment of male infertility by using combined LS and CoQ10 administration.

## 2. Materials and Methods

### Preparation of herbal extract

In this laboratory experimental study, to prepare the aqueous LS seed extract, the Moroccan traditional method was followed (‎17). Briefly, LS seeds were obtained from regional botanical shops, dried in the shade, and kept away from light (to prevent light-dependent reactions in seed cells) in closed containers until use. A mixture of 100-ml distilled water and 1 gr of powdered seeds was simmered for 10 min and left to be cooled for 15 min. It was then percolated by using a Millipore filter (Millipore 0.2, St. Quentin en Yvelines, France). The filtered extract was freeze-dried and the desired concentrations were prepared as fresh daily samples just before the treatment.

### Drug

Purified CoQ10 powder (Pure bulk, US) was used in this study.

### Animals and experimental design

Eighty three-months-old NMRI male mice (30–35 gr) were obtained from the Pasture Institute (Tehran, Iran). The animals were maintained in standard cages under controlled temperature (20–22∘C), humidity (25–30%), and a 12-hr light/dark cycle, with ad libitum access to standard commercial food and freshwater. Mice were handled with humane care according to the guidelines for the care and use of laboratory animals. Anesthetized animals underwent surgery and were sacrificed after the period of treatment. According to the previous studies (‎18,‎ 19), the animals were divided into the following groups of 10 mice in each group: control group (treated with water, 0.2 ml via gavage), CoQ10-treated groups (treated with 200, 300, and 400 mg/kg/body weight of CoQ10 via gavage), and LS-treated groups (treated with 200, 400, 600 mg/kg/body weight of LS seed extract via gavage). The treatments were performed daily for 2 wk and targeted second half and late phase of spermatogenesis. After obtaining the results, at this step, 600 mg/kg/body weight of LS seed extract and 200 mg/kg/body weight of CoQ10 exhibited the highest improving effects on studied indices, therefore, a group (treated with 600 mg/kg/body weight of LS seed extract + 200 mg/kg/body weight CoQ10 via gavage) was designed as combined group which received the treatment daily for 2 wk.

### Evaluation of male sexual behavior

The evaluation of male sexual behavior was carried out using the method described by Schrader and Lemasters (‎20). In brief, sexual behavior (sniffing, following, mounting, and coupling) of male mice was evaluated 2 wk after the treatment by using receptive female mice in the male's home cage. The cage contained wood chips, food, and water. To prepare the host for male sexual behavior examination, the female mice were injected with estradiol benzoate (50 µg/kg) and progesterone (500 µg/kg) for three consecutive days and 6 hr before the test, respectively. For the study of sexual behavior, the control and the experimental groups were separately placed in a cage with a sexually experienced male and a receptive female. For each behavior, the delay in the onset of behavior and the number of sniffing, following, mounting, and coupling behaviors were measured for 60 min, and the results of the treatment group were compared with the control group. Behavioral parameters were videotaped at the time and analyzed after the experimental procedure had been completed.

### Hormone assay

Serum levels of follicle-stimulating hormone (FSH), luteinizing hormone (LH), and testosterone were determined in duplicate by using solid-phase enzyme-linked immunosorbent assay (ELISA) kits (Fine Test, China) with sensitivities of < 1.4 ml U/ml, < 0.3 ng/ml, and < 1.8 ng/ml, respectively. Intra-assay and inter-assay coefficients of variation were < 10%, according to the competitive binding principle and the manufacturer's instructions.

### Evaluation of epididymal sperm count

The animals were anesthetized with a combination of ketamine and xylazine, then the testis and epididymis were removed and analyzed under the light microscope (Olympus IX70, Tokyo). Epididymis spermatozoa were retrieved, diluted in 1 ml of normal saline, and incubated for 15 min (37∘C). The sperm quality was determined by Sperm Class AnalyzerⓇ (SCA) CASA system (HTF, Tehran, Iran) containing a bright field microscope (Nikon E-200, Japan), a digital camera (Canon, Japan), and a computer with SCAⓇ software (Canon, USA). At least 400 sperm cells were captured and analyzed for each sample (‎21).

### GnRH gene expression analysis 

Mouse hypothalamus total RNA was extracted according to the manufacturer's protocol from freshly harvested tissue using the TRIzol reagent (Invitrogen, USA). Next, after being crushed physically by the sampler and chemically by 1 ml of TRIzol, the sample was mixed with 200 ul of chloroform, shaken vigorously for 30 sec, and then centrifuged at 15,000 g for 5 min at –10 to –7∘C.

The upper aqueous phase containing DNA and RNA and with 200 ul of the volume was transferred to a sterile RNase-free tube. The total RNA was then precipitated by adding 500 ul of cold isopropanol, shaking for 5 min, and centrifuging at 1,200 g for 10 min at 4 to 8∘C, followed by two washing steps by 75% ethanol. After the evaporation of the alcohol, the pellet containing RNA was dissolved in distilled water. The RNA product and its purity were evaluated by spectrometry analysis. RNA electrophoresis on a 1% agarose gel was done to assure the absence of RNA degradation. After DNase treatment to remove genomic contamination, 2.0 μg of total RNA from each sample was reverse transcribed by using the First Strand cDNA Synthesis Kit (Fermentas, USA) according to manufacturer's instructions using random hexamer primers.

The mRNA expression of the GnRH gene was evaluated using real-time polymerase chain reaction (PCR) by SYBR Green Master Mix (Qiagen Fast Start Universal, SYBR Green Master Mix) under the following conditions: denaturation at 95∘C for 10 sec, annealing at 60∘C for 10 sec, and extension at 72∘C for 20 sec with 40 repeated thermal cycles. PCR reactions were carried out in triplicate and the expression of all mRNAs was calculated using the LinReg PCR software to see relative changes in gene expression by using the β2-microglobulin (*B2M*) gene as an internal control because of its high stability. Table I shows the cDNA primers used in this experiment. The primers used in this study were synthesized by Sigma-Aldrich (St. Louis, MO, USA). The primers were evaluated using primer BLAST (http://blast.ncbi.nlm.nih.gov/Blast.cgi) .

**Table 1:** Primer sequences for GnRH and housekeeping genes


**Primer name**	**Primer sequence**	**Annealing temperature (∘C)**	**Cycle**	**Product size**
**GnRH-f**	CCAGCCAGCACTGGTCCT	60	94 bp
**GnRH-r**	CCACCTCCTTGGCCCATCTCTT		
**B2M-f**	ACTGAGACTGATACATACGCCTGC	58	40	90 bp
**B2M-r**	GCTTGATCACATGTCTCGATCCCA			
GnRH-f: Gonadotropin-releasing hormone‎ forward, GnRH-r: Gonadotropin-releasing hormone‎ reverse, B2M-f: Βeta 2-Microglobulin forward, B2M-r: Βeta 2-Microglobulin reverse				

### Ethical considerations 

This study was conducted in accordance with the guidelines of the Medical Ethics Committee on the use and care of laboratory animals. All investigations conformed to the ethical and humane principles of research and were approved by the research ethics committee of the Tehran Islamic Azad University of Medical Sciences (Code: IR.IAU.TMU.REC.1398.095).

### Statistical analysis

Data are presented as mean ± SEM and were analyzed using the unpaired *t* test and one-way analysis of variance followed by Tukey's post hoc test. P-values < 0.05 were considered as statistically significant. Statistical analysis was performed using GraphPad Prism 7 software (GraphPad Software Inc., San Diego, USA) and SPSS20 (SPSS Inc., Chicago, USA).

## 3. Results

### Effect of LS on sexual behavior

**Figure 1:**
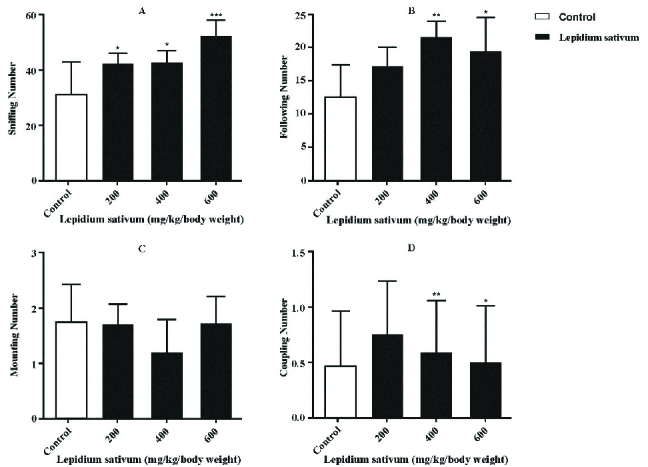
The effects of LS on sniffing (A), following (B), mounting (C), and coupling (D) number in *LS*-treated and control groups. *p < 0.05, **p < 0.01, and ***p < 0.05, Significant difference compared with the control group.

To evaluate the sexual behavior of the experimental and control groups of mice, receptive female mice were placed in the males' home cages. Our observation revealed that the numbers of sniffing (p = 0.0007, Figure 1A) and following (p = 0.0010, Figure 1B) behaviors were significantly higher in the LS-treated groups (400 and 600 mg/kg/body weight) than the control group, while there was no significant difference in the numbers of mounting (p = 0.3635, Figure 1C) and coupling (p = 0.4178, Figure 1D) behaviors between the experimental and control groups.

### Effect of CoQ10 on sexual behavior

**Figure 2:**
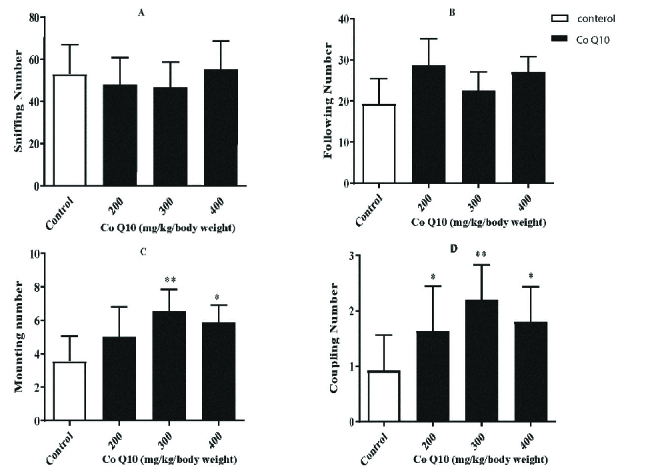
The effects of CoQ10 on sniffing (A), following (B), mounting (C), and coupling (D) number in CoQ10-treated and control groups. *p < 0.05 and **p < 0.01, Significant difference compared with the control group.

The numbers of sniffing (p = 0.6003, Figure 2A) and following (p = 0.7602, Figure 2B) behaviors did not show a significant difference compared to the control group. The monitoring of sexual behavior in the experimental and control groups showed that CoQ10-treated groups (300 and 400 mg/kg/body weight) exhibited significantly more frequent mounting (p = 0.017, Figure 2C) and coupling (p = 0.0006, Figure 2D) behaviors than the control group.

### Combination effect of CoQ10 and LS on sexual behavior

**Figure 3:**
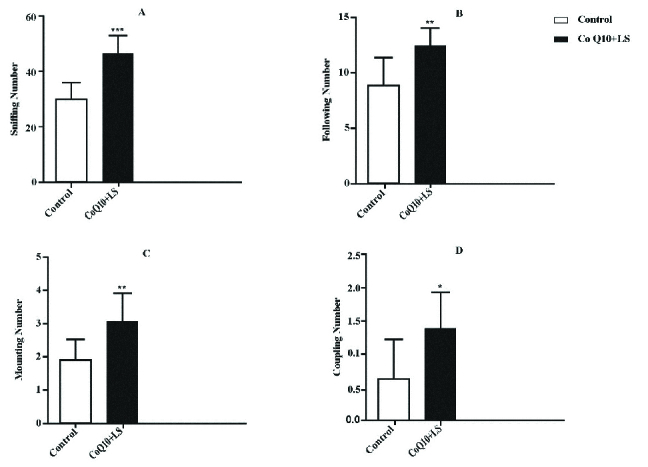
The effects of co-treatment of CoQ10 (200 mg/kg/body weight) and LS (600 mg/kg/body weight) on sniffing (A), following (B), mounting (C), and coupling (D) number in CoQ10 + LS-treated and control groups. *p < 0.05, **p < 0.01, and ***p < 0.05, Significant difference compared with the control group.

Compared to the data obtained from the control group, the co-treatment of CoQ10 (200 mg/kg/body weight) and LS (600 mg/kg/body weight) caused a significant increase in all aspects of sexual behaviors: sniffing (p = 0.0022, Figure 3A), following (p = 0.0082, Figure 3B), mounting (p = 0.0124, Figure 3C), and coupling (p = 0.0267, Figure 3D) behaviors.

### Co-treatment effect of CoQ10 and LS on the serum levels of testosterone, LH, and FSH

**Figure 4:**
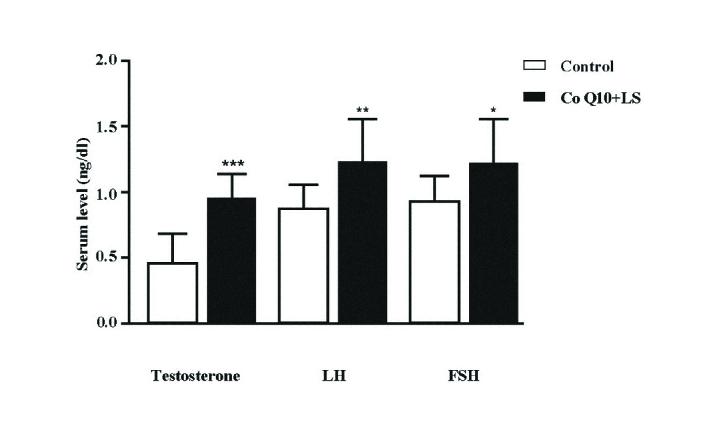
Co-treatment effect of CoQ10 (200 mg/kg/body weight) and LS (600 mg/kg/body weight) on the serum levels of testosterone, LH, and FSH. *p < 0.05, **p < 0.01, and ***p < 0.05, Significant difference compared with the control group.

The mean values of serum testosterone (p = 0.0011), LH (p = 0.0062), and FSH (p = 0.0001) were revealed to be significantly higher for the co-treatment group than the control group (Figure 4).

### Co-treatment effect of CoQ10 and LS on semen parameters

The co-treatment of CoQ10 and LS intensified sperm parameters. The percentage of sperm viability increased significantly compared to the pretreatment sperm viability (p = 0.03). The total sperm motility increased significantly by 5% (p = 0.001) after the co-treatment. There was no significant difference in the total sperm count (p = 0.6532) and the number of healthy sperms between the control and co-treated groups (p = 0.7322) (Table II).

### Expression analysis of GnRH gene

**Figure 5:**
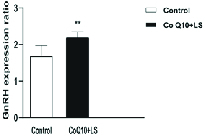
Co-treatment effect of CoQ10 (200 mg/kg/body weight) and LS (600 mg/kg/body weight) on the *GnRH* gene expression level compared with the control group. **p < 0.01, Significant difference compared with the control group.

Based on the results obtained from real-time PCR data analysis, the co-treatment of CoQ10 (200 mg/kg/body weight) and LS (600 mg/kg/body weight) significantly increased the relative expression level of GnRH gene compared with the control group (p = 0.0016) (Figure 5).

**Table 2:** Co-treatment effect of CoQ10 and LS on the sperm count, motility, viability, and morphology in NMRI mice


	**Control**	**CoQ10 and LS**
**Sperm count (million/ml)**	6.95 ± 0.9	7.4 ± 1.1
**Sperm total motility (%)**	40.75 ± 3.3	45.8 ± 3.2**
**Sperm viability (%)**	22.7 ± 2.9	26.2 ± 3.1*
**Sperm normal morphology (%)**	74.8 ± 4.7	76.8 ± 6.9
Data are presented as Mean ± SEM. *Significant difference compared with the control group (*p < 0.05 and **p < 0.01). Data were analyzed using unpaired *t* test. LS: *Lepidium sativum*		

## 4. Discussion

Our findings indicate that while LS treatment increases the sniffing and following behaviors, CoQ10 treatment enhances the mounting and coupling behaviors in mice. Interestingly, a co-treatment of CoQ10 and LS led to enhancement in all aspects of sexual behaviors, that is, sniffing, following, mounting, and coupling behaviors, as well as sperm quantity and quality accompanied by an increase in serum testosterone, LH, and FSH levels which was partly caused by increased GnRH gene expression level. The findings clearly show that CoQ10 with LS can prevent infertility or play a big role in male infertility treatment.

Indeed, male infertility accounts for 30–50% of infertility cases. Generally, male fertility depends on the quality, motility, and sperms morphology, therefore any disorder in these factors can lead to a dysfunction in reproductive system (‎2). In line with our findings, it has been shown that reduced antioxidant capacity caused by decreased cellular CoQ10 function or content can lead to an increase in free radicals' adverse effects on sexual behavior and fertility (‎22). CoQ10 is an androgenic-soluble benzoquinone compound found throughout the body in cell membranes, especially in the mitochondrial membranes, and is abundant in the heart, lungs, and adrenal glands and has improving effects on male reproductive organs (‎23). CoQ10 induces antioxidant activity in rat testes (‎24). In addition, it has been reported that CoQ10 supplementation can boost fertility factors such as sperm count and motility (‎16) and elevates the sexual hormones levels and spermatogenesis in rats (‎25).

Many medicinal herbs have been traditionally administrated to treat infertility due to their potential beneficial effects on male fertility parameters (‎2). One strategy for the evaluation of couples with male infertility is the identification of sexual behavior parameters. LS is one of the most popular herbs used to elevate sexual pleasure and sexual behaviors (‎‎11). It has been reported to increase dopamine and acetylcholine levels and regulate gamma-aminobutyric acid (GABA) secretion in rats, both of which are related to sexual behavior (‎26). Moreover, LS increases the blood supply and consequently erection via nitric oxide synthesis induction in the endothelial cells (‎‎‎27). LS can also enhance sex steroid levels in male and female reproductive system (‎‎28). Other therapeutic effects of LS on the rat reproductive system such as spermatogenesis and serum LH level enhancement as well as its antioxidant activity in mice have been identified by recent investigations (‎11). In accordance with previous studies, our results showed that the treatment of mice with 600 mg/kg/body of LS significantly improves some aspects of sexual behavior in male mice.

We have shown that a co-administration of LS and CoQ10 has a complementary effect on all aspects of sexual behavior compared to LS (or CoQ10) alone which improved only some aspects – not all – of sexual behavior. The hypothalamus secretory-neural axis and hypothalamus-pituitary-gonads (HPG) axis play important roles in sexual behavior control and gametogenesis through GnRH, LH, FSH, and sexual steroid hormone production. Any dysfunction in these axes can result in sexual desire disorder or infertility (‎‎‎29). Interestingly, our results revealed that co-treatment with CoQ10 and LS significantly induces the GnRH gene overexpression, which, in turn, elevates the FSH and LH levels. The elevated FSH and LH levels in turn causes an increase in serum testosterone level and sperm count, respectively. Our findings suggest that CoQ10 + LS supplementation has a strong regulatory influence on the HPG axis and stimulate the release of GnRH and LH from hypothalamus and pituitary gland, respectively. The results of the current study also suggest that the increase in motility and viability of sperms as well as sexual activity that followed co-treatment with CoQ10 and LS could be related to the changes in sex hormone levels. It has been shown that CoQ10 has beneficial effects on the reproductive hormones metabolism and secretion (‎‎30).

The fundamental effect of CoQ10 on sperm parameters may be mediated through multiple pathways. CoQ10 is concentrated within the mitochondrial midpiece of sperm and provides the energy for sperm movement (31). It can also have additive neuroprotective effects and reduces lipid peroxidation of the sperm membrane, which increases the membrane fluidity and motility (‎‎32). On the other hand, LS has antioxidant properties (‎8) and a positive impact on sexual behaviors and fertility (‎4,‎ 12). Taken together, these facts suggest that LS and CoQ10 can synergistically increase spermatogenesis, sex hormones production and release, and antioxidant activity. Accordingly, all aspects of sexual behavior in male mice can be enhanced by co-administration of LS and CoQ10 via inducing sexual hormones secretions. However, the exact cellular and molecular basis for the improving effects of LS and CoQ10 on male reproductive system of male mice remains unknown and further research focusing on LS and CoQ10 effects on HPG axis and investigating the cellular and molecular mechanisms involved in LS and CoQ10 effects on testicular tissue as well as sperm cells will reveal many mysteries behind their action on target cells and tissues.

## 5. Conclusion

Our results demonstrate the synergistic effects of CoQ10 and LS supplementation on the semen parameters, sperm function, and sexual behaviors. The improving effects of LS and CoQ10 on male reproductive system are at least in part mediated by their regulatory effects on HPG axis via inducing the GnRH gene overexpression and increasing of LH, FSH, and testosterone production or secretion.

##  Conflict of Interest 

The authors declare that they have no conflict of interest.
